# The potential to improve ascertainment and intervention to reduce smoking in Primary Care: a cross sectional survey

**DOI:** 10.1186/1472-6963-8-6

**Published:** 2008-01-11

**Authors:** Rachael L Murray, Tim Coleman, Marilyn Antoniak, Alexia Fergus, John Britton, Sarah A Lewis

**Affiliations:** 1 Division of Epidemiology & Public Health, University of Nottingham, Nottingham, UK; 2 Division of Primary Care, University of Nottingham, Nottingham, UK

## Abstract

**Background:**

Well established clinical guidelines recommend that systematic ascertainment of smoking status and intervention to promote cessation in all smokers should be a fundamental component of all health care provision. This study aims to establish the completeness and accuracy of smoking status recording in patients' primary care medical records and the level of interest in receiving smoking cessation support amongst primary care patients in an inner city UK population.

**Methods:**

Postal questionnaires were sent to all patients aged over 18 from 24 general practices in Nottingham UK who were registered as smokers or had no smoking status recorded in their medical notes.

**Results:**

The proportion of patients with a smoking status recorded varied between practices from 42.4% to 100% (median 90%). Of the recorded smokers who responded to our questionnaire (35.5% of the total), a median of 20.3% reported that they had not smoked cigarettes or tobacco in the last 12 months. Of respondents with no recorded smoking status, 29.8% reported themselves to be current smokers. Of the 6856 responding individuals thus identified as current smokers, 41.4% indicated that they would like to speak to a specialist smoking adviser to help them stop smoking. This proportion increased with socioeconomic disadvantage (measured by the Townsend Index) from 39.1% in the least deprived to 44.6% in the most deprived quintile.

**Conclusion:**

Whilst in many practices the ascertainment of smoking status is incomplete and/or inaccurate, failure to intervene appropriately on known status still remains the biggest challenge.

**Trial registration:**

Current Controlled Trials ISRCTN71514078.

## Background

The UK suffers a huge burden of premature mortality and morbidity as a direct result of tobacco use. The prevalence of cigarette smoking in 2005 was estimated at 24% of those aged over 16 in the UK [1]. Well established clinical guidelines in both the USA [2] and UK [3] recommend that systematic recording of smoking status and intervention to promote cessation in all smokers is highly cost-effective and should be a fundamental component of all health care provision. Previous studies indicate, however, that the recording of smoking status in primary care medical records is often inaccurate [4,5] and that it is probably updated infrequently [4,5]. This limits the utility of smoking status recorded in patients' medical records for either clinical practice or for determining smoking prevalence within practices [4,5]. Recently however, a new contract was introduced for UK general practitioners (GPs – family physicians) which remunerates them for recording the smoking status of patients in their medical records. As this contract has increased the frequency with which GPs ascertain patients' smoking status [6], the completeness and accuracy of smoking status data in such records may have improved.

Once smokers have been identified and brief advice to quit delivered, smokers who are interested in receiving further help with smoking cessation should be referred to trained smoking cessation advisors who provide effective support [7] and who, in the UK, are available through the National Health Service [8]. UK national survey data indicate, however, that in 2005 only 41% of smokers reported having received cessation advice in the previous 5 years, and although 72% of smokers reported that they wanted to quit smoking, only 8% had been referred to an NHS stop smoking service in the previous year [9]. This discrepancy suggests that only a small proportion of smokers may be receiving appropriate interventions in primary care.

In this paper, we compare smoking status recorded within primary care medical records with survey findings to provide a contemporary assessment of the completeness and accuracy of smoking status recorded in primary care medical records. We also estimate the level of interest in receiving support with stopping smoking amongst smokers who are general practice patients and determine how this varies with their socio-demographic characteristics.

## Methods

Data were collected from patients who were registered with 24 general practices in Nottinghamshire which participated in a cluster randomised controlled trial of a new approach to provision of smoking cessation services (ISRCTN71514078). In 2005, all 90 practices within three of the four Nottingham Primary Care Trusts with list sizes of 10,000 or less were contacted to request their participation in the study. 27 practices agreed, and 24 were randomly selected to participate in the trial to meet the requirements of our power calculation. However, two practices withdrew during the study and were replaced by two of the three unused practices (from the original 27), selected at random. Practices provided details of the number of registered patients aged 18 and over and searched computerised medical records for Read Codes indicating patients' smoking status. The Nottingham Ethics Committee approved the study.

Patients aged 18 or over whose medical record indicated either that they were i) a current smoker or ii) contained no record of smoking status were sent a short, self-completion questionnaire with a standard letter on headed paper from participating practices. The letter explained that questionnaire responses would be used to update patients' medical records and also sought written consent for these to be used by the University of Nottingham research team in a study aimed at helping smokers to quit. The questionnaire confirmed current smoking status by asking respondents about smoking in the last 12 months, frequency of smoking (every day, most days, occasionally or never), and amount smoked per day (≤10, 11–20, 21–30, 31–40, 41+). The questions asked have been used in previous primary care studies, having been administered by postal and personally delivered self-completion questionnaires [10-12]. The questionnaire also asked current smokers whether they would like to speak to a smoking cessation advisor to receive help or advice to quit smoking. A reminder letter was sent out to non-responders after three weeks, completed questionnaires were returned to practices and, with appropriate consent, these were collected by the research team.

Questionnaire data were entered into SPSS Version 14. We defined current smokers as those who smoked occasionally or more frequently. Townsend scores based on patient's postcodes were calculated from the 2001 census [13]. Townsend scores are based on unemployment, car ownership, overcrowding and tenure, and this measure of deprivation has been found to explain variations in health measures and adhere closely to the concept of material disadvantage [14]. The proportion with a smoking status recorded, the proportions of recorded smokers who were not smoking (i.e. misclassified as smokers) and of self-reported smokers with no record of this in their medical records, and the proportion wanting to speak to an advisor, were calculated at the practice level, and presented as the median and range because the distributions of some of these data were skewed. Spearman correlation analysis was used to assess the correlation between the proportion with a smoking status and the proportion of patients misclassified as smokers. The effect of individual characteristics such as age, sex and Townsend Index on whether individuals responded to the questionnaire, were misclassified as smokers, and whether smokers wanted help to quit, was analysed at the individual level using logistic regression, and robust standard errors to allow for clustering by practice using STATA release 9.0; STATA Corp., College Station, TX.

## Results

### Response to questionnaire and recording of smoking status

Within the 24 participating general practices there were 87,861 patients aged 18 or over, of whom 23,044 were recorded as smokers, 52,629 as non-smokers and 12,188 had no record of smoking status in their medical records. Of 35232 questionnaires dispatched, the proportion of patients returning the questionnaire and giving signed consent for their information to be shared with the research team, varied between practices from 13.9% to 41.1% (median 33.2%). Respondents recorded as smokers in their medical records were more likely to respond than those with no smoking status recorded [35.5% (8176/23044) and 24.2% (2951/12188) respectively], and males and younger patients were less likely to respond to the questionnaire (Table 1).

**Table 1:** Questionnaire response rates and numbers of self reported smokers

	NUMBER SENT	NUMBER (%) RETURNED	NUMBER OF SELF-REPORTED CURRENT SMOKERS
TOTAL	35232	11127(31.6)	6856
Practice median (range)	1312 (432–2985)	33.2% (13.9–41.4%)	258 (52–766)
			
SMOKER	23044	8176 (35.5)	5943
NO STATUS	12188	2951 (24.2)	913
			
MALE	20040	5839 (29.1)	3515
FEMALE	15192	5288 (34.8)	3340
			
AGE			
<= 30	9965	2161 (21.7)	1344
31–40	8176	2187 (26.7)	1430
41–50	6635	2205 (33.2)	1440
51–60	5007	2052 (41.0)	1305
61 +	5449	2522 (46.3)	1337

**Figure 1:**
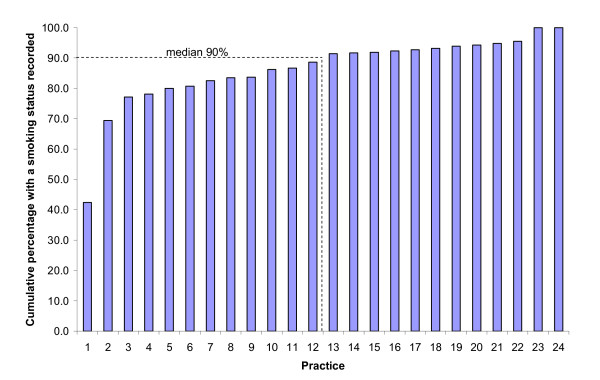
Cumulative distribution of the percentage of patients in a practice with a smoking status recorded.

The proportion of patients with smoking status recorded varied between practices from 42.4% to 100% (median 90.0%, see Figure 1). The proportion of responding patients recorded as smokers who denied tobacco use in the previous 12 months varied between 6.3% and 58.1% across practices (median 20.3%). There was no correlation between the proportion of patients with a smoking status recorded and the proportion of patients who were recorded as smokers but denied tobacco use in the previous 12 months (Spearmans r = -0.14). The proportion of patients with no record of smoking status who were in fact self reported current smokers varied from 5.7% to 60.2% across practices (median 29.8%).

The proportion of patients misclassified as smokers in their medical record was unrelated to gender, but did vary with age. In those aged 30 or below, 13.1%, of patients recorded as smokers in their medical record reported not smoking in the past year on questionnaires and this increased to 31.7% in those aged over 61.

### Smokers' interest in support with stopping smoking

Of the 6856 respondents who were current smokers, 2840 (41.4%) indicated that they would like to speak to a specialist smoking cessation advisor to help them stop smoking (Table 2). This varied between practices from 30.6% and 51.8% (median 39.8%). Individuals who were previously recorded as smokers tended to be more likely to want to speak to a cessation adviser than those who previously had no smoking status recorded (42.7% and 33.4% respectively).

**Table 2:** Number of current smokers who would like support to quit smoking

	Number of smokers who responded	Number who wanted help to quit	%	Adjusted odds ratios (95% Cl)
Total	6856	2840	41.4	
				
Males	3516	1430	40.7	1
Females	3340	1410	42.2	1.07 (.97–1.17)
				
AGE				
<= 30	1344	447	33.4	1
31–40	1430	674	47.1	1.82 (1.55–2.13)
41–50	1440	680	47.2	1.83 (1.45–2.31)
51–60	1305	583	44.7	1.63 (1.34–1.99)
61 +	1337	456	34.1	1.05 (.89–1.25)
				
Townsend quintile 1	1373	537	39.1	1
Townsend quintile 2	1343	530	39.5	1.00 (.81–1.23)
Townsend quintile 3	1335	552	41.3	1.09 (.93–1.29)
Townsend quintile 4	1362	576	42.3	1.12 (.89–1.40)
Townsend quintile 5	1354	604	44.6	1.26 (1.01–1.56)

*Townsend quintile 1 = <= -1.60

*Townsend quintile 2 = -1.60 to -0.49

*Townsend quintile 3 = -0.49 to 3.03

*Townsend quintile 4 = 3.03 to 5.09

*Townsend quintile 5 = 5.09 +

**Townsend data for 84 consenting smokers was unavailable

Interest in discussion of support did not vary with gender (40.7% and 42.2%, for men and women respectively) but did vary with age and economic disadvantage. Those aged between 31 and 50 were most likely to want to speak to an advisor and the oldest and youngest age groups were least likely to desire this (33.4% and 34.1% respectively). Smokers' reported desire to talk with smoking cessation advisors increased linearly with economic disadvantage (measured by Townsend index) such that demand for support was highest (44.6%) from the most disadvantaged and lowest (39.1%) from the least socially disadvantaged groups.

## Discussion

Our study demonstrates that, in 2005, practices in our study had a recording of smoking status in the primary care medical record for, on average, 90% of registered patients, but this was probably not accurate in about 20% of cases. Additionally, amongst smokers who responded to questionnaires sent from their general practitioners, over 41% were interested in talking to a smoking cessation advisor to obtain support with stopping smoking and interest was highest amongst the most economically deprived smokers.

Smoking is still the biggest avoidable cause of death and disability in the UK and intervening to help smokers quit is highly cost-effective. Authoritative clinical guidelines recommend that the ascertainment of smoking status and delivery of brief advice to stop with further support for smokers interested in quitting should be a routine and systematic component of all medical consultations [3,15,16]. The findings of this study indicate that, although the ascertainment of smoking status in primary care is apparently high, these data are relatively inaccurate and more regular updating of smoking status records might increase the numbers of opportunities which health professionals use to intervene and promote smoking cessation.

To our knowledge, this study is the first to systematically contact large numbers of smokers living in a large, relatively-deprived urban area and ascertain their interest in engaging with smoking cessation support. This systematic approach within a defined population allows estimates of smokers' desire for support with smoking cessation to be made. The limitations of the study include the fact that participation was relatively low, which is likely to be partly attributable to inaccuracies in addresses on practices' registers, and that ethical constraints dictated that the research team obtained signed consent from questionnaire respondents before their data could be used for research purposes. As not all respondents gave their consent for their information to be used in this way, this will have lowered the response rate. We have assumed that the smoking status reported by questionnaire respondents was reliable, since questionnaire data obtained by similar means in previous studies [4,5] have been found to be accurate [17], and informing recipients that their responses would be used to update their medical records should, if anything, have improved the validity of responses. It is also possible that there may have been selection bias in the practices that took part, for example, they may have had a greater interest in smoking cessation than others.

Although 41% of smokers who responded to the questionnaire reported that they would like to speak to a smoking cessation advisor, this figure is almost certainly an overestimate of the true proportion. If we conservatively presume that all those who wanted help to quit responded, then the true denominator would be all current smokers who were sent a questionnaire, which we estimate, based on the accuracy of smoking status recording found in our study, to be 20,521, reducing the proportion wanting to speak to an adviser to 13.8%. Nevertheless, between April 2004 and March 2005, just over 500,000 smokers set quit dates using English NHS stop smoking services [18] and, as this represents less than 5% of English smokers, our findings suggest that there is considerable interest in speaking to cessation advisors, and potentially, receiving cessation support amongst smokers that is not currently translated into their use of NHS stop smoking services. The challenge for the UK National Health Service is to find ways of engaging smokers who are interested in talking to smoking cessation advisors and receiving support with stopping smoking and encouraging them to access such support. In particular, our results throw into question the reluctance of many GPs to raise the topic of smoking due to concern of negative responses from their patients [19], and suggests that this attitude results in missed opportunities to provide help and advice to smokers who would welcome this. Study findings suggest that the most economically disadvantaged smokers who suffer from the greatest smoking-related morbidity [20] are also the most interested in receiving support. It is important to ensure that this group is appropriately assisted, possibly by using novel methods of 'marketing' NHS stop smoking services to this group.

The proportion of primary care patients in our inner city practices whose records included a note of smoking status (median 90%) was higher than in previous studies (73.4% and 76%) [4,5] and this more comprehensive recording could be due to the introduction of the 2004 general practice contract which has increased rates of smoking status ascertainment [6].

However, we have no data from study practices during the period before the contract was introduced to compare our findings with and recording rates may be higher for other reasons. Higher rates of smoking status recording amongst women and older people have been observed previously [5], and are probably influenced by these patients' higher general practice consultation rates [21].

Nevertheless, across practices, an average of 20% of individuals recorded in their medical records as smokers were not currently smoking; whilst this may be an overestimate of the true figure for our study population if smokers who had successfully quit were more likely to return the questionnaire, it is also possible that offering support to stop smoking may have encouraged more current smokers to return the questionnaire. This level of accuracy of recorded smoking status is no better than that found in earlier studies. In the late 1990s, Wilson *et al.*[5] found that around 18% of patients recorded as smokers in general practice medical records reported in postal questionnaires that they were not. Our rate is very similar, and moreover, we found a large variation between practices in the proportion of smokers who were misclassified such that in one practice this reached 58.1%. We also observed that the proportion misclassified as smokers increases with age, suggesting that once patients' smoking status has been ascertained, it is not routinely updated, so that the accuracy of this information reduces as time passes. A previous study found that although 99% of GPs record smoking status when patients first join their practices, only 57% claim to routinely update this information [3] and our findings may reflect this. Nevertheless, we found no correlation between the level of recording and misclassification suggesting that high ascertainment of smoking status among practices in our sample was not necessarily at the expense of accuracy, and that both may be achieved.

At the time of our study, the general practice contract rewarded GPs for any record of smoking status that patients' records contained, irrespective of when this was obtained, but revisions to this (introduced in 2007) will result in GP's only being paid for ascertainment of smoking status that has occurred within the previous 15 months and this could generate more frequent updating of primary care smoking status records, enhancing their validity. A potential avenue for future research could ascertain whether these measures are effective in improving validity of this data.

## Conclusion

We have found that data on smoking status recorded in patients' primary care medical records contains inaccuracies which render it inappropriate for either effective health planning or research purposes. However, failure to intervene appropriately on known status still remains the biggest challenge.

Recent changes in general practitioners' contractual arrangements may improve the validity of these data and further monitoring of data validity after these are introduced is warranted. More importantly, a significant minority of smokers are interested in talking to smoking cessation advisors about receiving support and help with stopping smoking, but only a fraction of these actually try to stop smoking with the support of NHS stop smoking services. Engaging more of these 'interested' smokers in attempts to achieve smoking cessation is an important task which, if successful, could promote significant health gain by impacting on smoking rates in the UK.

## Competing interests

The author(s) declare that they have no competing interests.

## Authors' contributions

RLM collected and analysed data, and drafted the manuscript. SAL, TC and JB designed the study, supervised data collection and analysis, and commented on the manuscript. AF and MA collected the data and commented on the manuscript. All authors read and approved the final version of the manuscript. RLM is guarantor.

Funding: British Heart Foundation. The study was designed, conducted, analysed and interpreted independently of all funding sources.

## Pre-publication history

The pre-publication history for this paper can be accessed here:


